# Diaqua­bis­(5-carb­oxy-2-propyl-1*H*-imidazole-4-carboxyl­ato-κ^2^ *N* ^3^,*O* ^4^)cadmium *N*,*N*-dimethyl­formamide disolvate

**DOI:** 10.1107/S1600536811050264

**Published:** 2011-11-30

**Authors:** Shao-Wei Tong, Shi-Jie Li, Wen-Dong Song, Dong-Liang Miao, Jing-Bo An

**Affiliations:** a College of Food Science and Technology, Guangdong Ocean University, Zhanjiang 524088, People’s Republic of China; b School of Enviroment Science and Engineering, Donghua University, Shanghai 200051, People’s Republic of China; c College of Science, Guangdong Ocean University, Zhanjiang 524088, People’s Republic of China

## Abstract

In the title complex, [Cd(C_8_H_9_N_2_O_4_)_2_(H_2_O)_2_]·2C_3_H_7_NO, the six-coordinate Cd^II^ ion is in a slightly distorted octa­hedral environment, defined by two O atoms from two coordinated water mol­ecules and two carboxyl­ate O atoms and two N atoms from two *N*,*O*-bidentate 5-carb­oxy-2-propyl-1*H*-imidazole-4-carboxyl­ate ligands. In the crystal, complex mol­ecules and dimethyl­formamide solvent mol­ecules are linked by O—H⋯O and N—H⋯O hydrogen bonds into a two-dimensional supra­molecular structure. The propyl groups of the ligands are disordered over two conformations with refined occupancies of 0.680 (7) and 0.320 (7).

## Related literature

For our past work based on the H_3_PIDC (2-propyl-imidazol-4,5-dicarb­oxy­lic acid) ligand, see: Fan *et al.* (2010 ▶); Li, Song, Miao, Tong *et al.* (2011 ▶); Li, Miao *et al.* (2010 ▶); Li, Yan *et al.* (2010 ▶); Song *et al.* (2010 ▶); He *et al.* (2010 ▶); Yan *et al.* (2010 ▶). For our past work based on the H_3_EIDC (2-ethyl-1*H*-imidazol-4,5-dicarb­oxy­lic acid) ligand, see: Li, Ma *et al.* (2011 ▶); Li, Song, Miao, Hu *et al.* (2011 ▶). 
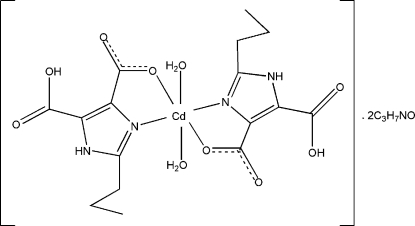


## Experimental

### 

#### Crystal data

[Cd(C_8_H_9_N_2_O_4_)_2_(H_2_O)_2_]·2C_3_H_7_NO
*M* *_r_* = 688.97
Orthorhombic, 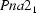

*a* = 16.6040 (14) Å
*b* = 9.8516 (8) Å
*c* = 18.4154 (16) Å
*V* = 3012.3 (4) Å^3^
*Z* = 4
Mo *K*α radiation
μ = 0.79 mm^−1^
*T* = 295 K
0.27 × 0.24 × 0.21 mm

#### Data collection

Rigaku/MSC Mercury CCD diffractometer
Absorption correction: multi-scan (*REQAB*; Jacobson, 1998 ▶) *T*_min_ = 0.815, *T*_max_ = 0.851
16187 measured reflections
4421 independent reflections
3111 reflections with *I* > 2σ(*I*)
*R*_int_ = 0.046

#### Refinement

*R*[*F* ^2^ > 2σ(*F* ^2^)] = 0.035
*wR*(*F* ^2^) = 0.079
*S* = 1.00
4421 reflections
444 parameters
233 restraints
H atoms treated by a mixture of independent and constrained refinement
Δρ_max_ = 0.41 e Å^−3^
Δρ_min_ = −0.39 e Å^−3^
Absolute structure: Flack (1983 ▶), 1285 Friedel pairs
Flack parameter: −0.04 (4)

### 

Data collection: *RAPID-AUTO* (Rigaku, 1998 ▶); cell refinement: *RAPID-AUTO*; data reduction: *CrystalStructure* (Rigaku/MSC, 2002 ▶); program(s) used to solve structure: *SHELXS97* (Sheldrick, 2008 ▶); program(s) used to refine structure: *SHELXL97* (Sheldrick, 2008 ▶); molecular graphics: *SHELXTL* (Sheldrick, 2008 ▶); software used to prepare material for publication: *SHELXTL*.

## Supplementary Material

Crystal structure: contains datablock(s) I, global. DOI: 10.1107/S1600536811050264/pk2369sup1.cif

Structure factors: contains datablock(s) I. DOI: 10.1107/S1600536811050264/pk2369Isup2.hkl

Additional supplementary materials:  crystallographic information; 3D view; checkCIF report

## Figures and Tables

**Table 1:** Selected bond lengths (Å)

Cd1—N4	2.262 (4)
Cd1—N2	2.262 (4)
Cd1—O2*W*	2.325 (6)
Cd1—O1*W*	2.322 (5)
Cd1—O4	2.356 (5)
Cd1—O8	2.357 (5)

**Table 2:** Hydrogen-bond geometry (Å, °)

*D*—H⋯*A*	*D*—H	H⋯*A*	*D*⋯*A*	*D*—H⋯*A*
O2—H2⋯O3	0.82	1.69	2.460 (6)	155
O6—H6⋯O7	0.82	1.64	2.453 (6)	174
O1*W*—H1*W*⋯O10	0.83 (2)	1.94 (2)	2.763 (6)	175 (9)
O1*W*—H2*W*⋯O5^i^	0.82 (2)	2.00 (4)	2.771 (6)	158 (9)
O2*W*—H3*W*⋯O1^ii^	0.80 (2)	2.02 (3)	2.787 (6)	161 (7)
O2*W*—H4*W*⋯O9	0.80 (2)	2.02 (3)	2.791 (6)	162 (8)
N1—H1*A*⋯O10^iii^	0.86	1.91	2.761 (6)	170
N3—H3*A*⋯O9^iv^	0.86	1.94	2.792 (6)	171

Symmetry codes: (i) 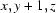; (ii) 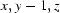; (iii) 
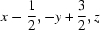
; (iv) 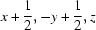.

**Fig. 1.**
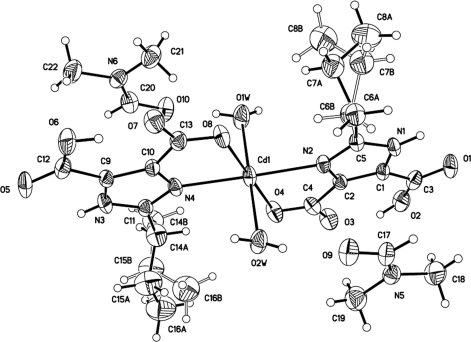
The structure of the title compound, non-H atoms are shown with 30% probability displacement ellipsoids.

## supplementary crystallographic information

### Comment

In recent years, structures containing metals and N-heterocyclic carboxylic acids have drawn increasing attention due to their fascinating structures and potential applications in many fields.  For instance, N-heterocyclic carboxylic acids H_3_IDC (imidazole-4,5-dicarboxylic acid) which can be deprotonated to form H_2_IDC^-^, HIDC^2-^ and IDC^3-^ anions under various pH conditions, have been broadly used to obtain a variety of metal-organic frameworks with novel structures and exceptional properties.  In our previous research, efforts have been focused on the design and synthesis of interesting metal organic complexes with derivatives of H_3_IDC, such as H_3_PIDC (2-propyl-imidazole-4,5-dicarboxylic acid) (Fan *et al.*, 2010; Li, Miao *et al.*, 2010; Li, Yan *et al.*, 2010; Li, Song, Miao, Tong *et al.*, 2011; He *et al.*, 2010; Song *et al.*, 2010; Yan *et al.*, 2010) and H_3_EIDC (2-ethyl-1*H*-imidazole-4,5-dicarboxylic acid) (Li, Song, Miao, Hu *et al.*, 2011; Li, Ma *et al.*, 2011). To continue our studies, we report the synthesis and structure of a new Cd(II) complex obtained from the H_3_PIDC ligand and cadmiun nitrate under hydrothermal conditions.

As shown in the Fig. 1, the title complex consists of one Cd^II^ ion, two mono-deprotonated H_2_PIDC ligands, two coordinated water molecules and two dimethylformamide solvent molecules. The Cd^II^ atom is six-coordinate in a slightly distorted octahedral geometry, connected with two N,O-bidentate ligands [Cd—O = 2.321 (5) Å and Cd—N = 2.262 (4) Å] and two coordinated water molecules [Cd—O = 2.356 (5) Å]. It is noted that the two imidazole rings are nearly coplanar.  In the crystal structure, the complex molecules and dimethylformamide solvent molecules are connected *via* hydrogen bonds (Table 1) into a two-dimensional supramolecular structure. The propyl groups of H_2_PIDC^-^ are disordered over conformations with refined occupancies of 0.679 (7):0.321 (7).

### Experimental

A mixture of Cd(CH_3_COO)_2_ (0.2 mmol, 0.046 g) and 2-propyl-1*H*-imidazole-4,5-dicarboxylic acid (0.2 mmol, 0.39 g) in 15 ml DMF was sealed in an autoclave equipped with a Teflon liner (25 ml) and then heated at 413 K for 3 days. Crystals of the title compound were obtained by slow evaporation of the solvent at room temperature.

### Refinement

H atoms of the water molecule were located in a difference Fourier map and refined subject to O—H distance restraints of 0.82 (1) Å, and *U*_iso_(H) = 1.5 *U*_eq_. The H···H distances within the water molecules were restraint to 1.30 (1) Å.  Carboxyl H atoms were located in a difference map but were refined as riding on the parent O atoms with O—H = 0.82 Å and *U*_iso_(H) = 1.5 *U*_eq_(O). Carbon and nitrogen bound H atoms were placed at calculated positions and were treated as riding on the parent C or N atoms with C—H = 0.96 (methyl), 0.97 (methylene) and N—H = 0.86 Å, *U*_iso_(H) = 1.2 or 1.5 *U*_eq_(C, N). The propyl groups of H_2_PIDC^-^ are split over two sites with refined occupancies of 0.679 (7):0.321 (7).

### Crystal data

**Table:**

[Cd(C_8_H_9_N_2_O_4_)_2_(H_2_O)_2_]·2C_3_H_7_NO	*F*(000) = 1416
*M**_r_* = 688.97	*D*_x_ = 1.519 Mg m^−^^3^
Orthorhombic, *P**n**a*2_1_	Mo *K*α radiation, λ = 0.71073 Å
Hall symbol: P 2c -2n	Cell parameters from 3600 reflections
*a* = 16.6040 (14) Å	θ = 1.4–28°
*b* = 9.8516 (8) Å	µ = 0.79 mm^−^^1^
*c* = 18.4154 (16) Å	*T* = 295 K
*V* = 3012.3 (4) Å^3^	Block, colourless
*Z* = 4	0.27 × 0.24 × 0.21 mm

### Data collection

**Table:**

Rigaku/MSC Mercury CCD diffractometer	4421 independent reflections
Radiation source: fine-focus sealed tube	3111 reflections with *I* > 2σ(*I*)
graphite	*R*_int_ = 0.046
ω scans	θ_max_ = 26.3°, θ_min_ = 2.2°
Absorption correction: multi-scan (*REQAB*; Jacobson, 1998)	*h* = −20→20
*T*_min_ = 0.815, *T*_max_ = 0.851	*k* = −11→12
16187 measured reflections	*l* = −22→11

### Refinement

**Table:**

Refinement on *F*^2^	Secondary atom site location: difference Fourier map
Least-squares matrix: full	Hydrogen site location: inferred from neighbouring sites
*R*[*F*^2^ > 2σ(*F*^2^)] = 0.035	H atoms treated by a mixture of independent and constrained refinement
*wR*(*F*^2^) = 0.079	*w* = 1/[σ^2^(*F*_o_^2^) + (0.020*P*)^2^ + 3.2*P*] where *P* = (*F*_o_^2^ + 2*F*_c_^2^)/3
*S* = 1.00	(Δ/σ)_max_ = 0.002
4421 reflections	Δρ_max_ = 0.41 e Å^−^^3^
444 parameters	Δρ_min_ = −0.39 e Å^−^^3^
233 restraints	Absolute structure: Flack (1983), 1285 Friedel pairs
Primary atom site location: structure-invariant direct methods	Flack parameter: −0.04 (4)

### Special details

**Table:**

Geometry. All e.s.d.'s (except the e.s.d. in the dihedral angle between two l.s. planes) are estimated using the full covariance matrix. The cell e.s.d.'s are taken into account individually in the estimation of e.s.d.'s in distances, angles and torsion angles; correlations between e.s.d.'s in cell parameters are only used when they are defined by crystal symmetry. An approximate (isotropic) treatment of cell e.s.d.'s is used for estimating e.s.d.'s involving l.s. planes.
Refinement. Refinement of *F*^2^ against ALL reflections. The weighted *R*-factor *wR* and goodness of fit *S* are based on *F*^2^, conventional *R*-factors *R* are based on *F*, with *F* set to zero for negative *F*^2^. The threshold expression of *F*^2^ > σ(*F*^2^) is used only for calculating *R*-factors(gt) *etc*. and is not relevant to the choice of reflections for refinement. *R*-factors based on *F*^2^ are statistically about twice as large as those based on *F*, and *R*- factors based on ALL data will be even larger.

### Fractional atomic coordinates and isotropic or equivalent isotropic displacement parameters (Å^2^)

**Table:**

	*x*	*y*	*z*	*U*_iso_*/*U*_eq_	Occ. (<1)
Cd1	0.61035 (2)	0.49830 (5)	0.52305 (9)	0.04966 (12)	
O1	0.4830 (3)	1.1346 (4)	0.5670 (3)	0.0656 (16)	
O2	0.5807 (3)	1.0620 (5)	0.6382 (3)	0.0668 (14)	
H2	0.5962	0.9882	0.6534	0.080*	
O3	0.6581 (3)	0.8521 (5)	0.6574 (3)	0.0677 (14)	
O4	0.6630 (3)	0.6406 (4)	0.6142 (3)	0.0614 (13)	
O5	0.7407 (3)	−0.1351 (4)	0.4749 (3)	0.0665 (16)	
O6	0.6439 (3)	−0.0594 (5)	0.4016 (3)	0.0674 (14)	
H6	0.6161	0.0092	0.3987	0.081*	
O7	0.5653 (3)	0.1490 (4)	0.3846 (3)	0.0614 (13)	
O8	0.5579 (2)	0.3569 (4)	0.4313 (3)	0.0572 (13)	
O1W	0.7047 (3)	0.5956 (4)	0.4459 (4)	0.0730 (16)	
H1W	0.748 (3)	0.554 (6)	0.440 (5)	0.110*	
H2W	0.718 (4)	0.675 (3)	0.442 (5)	0.110*	
O2W	0.5174 (3)	0.4033 (4)	0.6026 (3)	0.0662 (15)	
H3W	0.519 (4)	0.325 (3)	0.590 (4)	0.099*	
H4W	0.473 (2)	0.434 (6)	0.596 (5)	0.099*	
N1	0.4706 (3)	0.8811 (4)	0.4998 (3)	0.0429 (15)	
H1A	0.4358	0.9371	0.4826	0.051*	
N2	0.5401 (2)	0.6945 (4)	0.5161 (4)	0.0391 (11)	
N3	0.7518 (3)	0.1189 (5)	0.5425 (3)	0.0483 (17)	
H3A	0.7873	0.0636	0.5592	0.058*	
N4	0.6815 (2)	0.3026 (4)	0.5273 (4)	0.0418 (11)	
C1	0.5241 (3)	0.9076 (5)	0.5542 (3)	0.0398 (14)	
C2	0.5677 (3)	0.7909 (5)	0.5641 (3)	0.0396 (14)	
C3	0.5277 (4)	1.0437 (6)	0.5882 (4)	0.0517 (18)	
C4	0.6343 (4)	0.7584 (6)	0.6152 (4)	0.0527 (17)	
C5	0.4819 (3)	0.7508 (5)	0.4775 (3)	0.0414 (14)	
C6A	0.4334 (10)	0.686 (2)	0.4188 (5)	0.059 (3)	0.680 (7)
H6A	0.3842	0.7379	0.4119	0.071*	0.680 (7)
H6B	0.4183	0.5953	0.4338	0.071*	0.680 (7)
C7A	0.4787 (7)	0.6779 (11)	0.3461 (6)	0.076 (3)	0.680 (7)
H7A	0.5335	0.6477	0.3544	0.091*	0.680 (7)
H7B	0.4525	0.6126	0.3145	0.091*	0.680 (7)
C8A	0.4793 (9)	0.8133 (12)	0.3110 (7)	0.105 (4)	0.680 (7)
H8A	0.4993	0.8050	0.2623	0.158*	0.680 (7)
H8B	0.5134	0.8735	0.3380	0.158*	0.680 (7)
H8C	0.4255	0.8491	0.3098	0.158*	0.680 (7)
C6B	0.439 (2)	0.676 (4)	0.4189 (8)	0.065 (4)	0.320 (7)
H6C	0.3824	0.6690	0.4324	0.077*	0.320 (7)
H6D	0.4603	0.5846	0.4167	0.077*	0.320 (7)
C7B	0.4436 (13)	0.738 (3)	0.3427 (13)	0.080 (3)	0.320 (7)
H7C	0.4013	0.7008	0.3124	0.096*	0.320 (7)
H7D	0.4354	0.8358	0.3458	0.096*	0.320 (7)
C8B	0.5227 (14)	0.710 (3)	0.3096 (14)	0.105 (6)	0.320 (7)
H8D	0.5154	0.6838	0.2598	0.157*	0.320 (7)
H8E	0.5487	0.6379	0.3357	0.157*	0.320 (7)
H8F	0.5556	0.7902	0.3118	0.157*	0.320 (7)
C9	0.6991 (3)	0.0907 (5)	0.4879 (3)	0.0409 (15)	
C10	0.6547 (3)	0.2079 (5)	0.4781 (3)	0.0399 (14)	
C11	0.7400 (3)	0.2446 (5)	0.5661 (3)	0.0434 (15)	
C12	0.6953 (4)	−0.0449 (6)	0.4524 (4)	0.0501 (17)	
C13	0.5881 (4)	0.2408 (6)	0.4287 (4)	0.0467 (16)	
C14A	0.7785 (8)	0.3070 (14)	0.6317 (5)	0.075 (3)	0.680 (7)
H14A	0.7634	0.4018	0.6353	0.091*	0.680 (7)
H14B	0.8367	0.3017	0.6276	0.091*	0.680 (7)
C15A	0.7501 (10)	0.2293 (14)	0.7006 (7)	0.108 (3)	0.680 (7)
H15A	0.6917	0.2293	0.7027	0.129*	0.680 (7)
H15B	0.7682	0.1358	0.6982	0.129*	0.680 (7)
C16A	0.7827 (10)	0.2939 (17)	0.7661 (6)	0.132 (4)	0.680 (7)
H16A	0.7636	0.2465	0.8083	0.199*	0.680 (7)
H16B	0.7653	0.3867	0.7681	0.199*	0.680 (7)
H16C	0.8404	0.2906	0.7648	0.199*	0.680 (7)
C14B	0.7994 (14)	0.320 (3)	0.6113 (10)	0.068 (4)	0.320 (7)
H14C	0.7917	0.4168	0.6047	0.081*	0.320 (7)
H14D	0.8536	0.2976	0.5955	0.081*	0.320 (7)
C15B	0.7901 (14)	0.284 (3)	0.6922 (12)	0.093 (4)	0.320 (7)
H15C	0.7966	0.1874	0.6990	0.112*	0.320 (7)
H15D	0.8312	0.3305	0.7203	0.112*	0.320 (7)
C16B	0.7095 (15)	0.327 (3)	0.7171 (14)	0.113 (6)	0.320 (7)
H16D	0.7064	0.3180	0.7689	0.169*	0.320 (7)
H16E	0.6693	0.2702	0.6949	0.169*	0.320 (7)
H16F	0.7003	0.4196	0.7037	0.169*	0.320 (7)
O9	0.3702 (2)	0.5396 (4)	0.6089 (3)	0.0675 (14)	
N5	0.3911 (3)	0.7234 (5)	0.6798 (3)	0.0514 (13)	
C17	0.3557 (3)	0.6572 (5)	0.6270 (3)	0.0559 (19)	
H17A	0.3162	0.7031	0.6009	0.067*	
C18	0.3685 (3)	0.8631 (5)	0.6949 (3)	0.080 (2)	
H18A	0.3248	0.8892	0.6637	0.119*	
H18B	0.4138	0.9215	0.6864	0.119*	
H18C	0.3519	0.8709	0.7447	0.119*	
C19	0.4499 (5)	0.6607 (9)	0.7254 (5)	0.087 (3)	
H19A	0.4500	0.5645	0.7170	0.131*	
H19B	0.4371	0.6784	0.7753	0.131*	
H19C	0.5022	0.6971	0.7145	0.131*	
O10	0.8514 (2)	0.4647 (4)	0.4356 (3)	0.0676 (15)	
N6	0.8302 (3)	0.2882 (5)	0.3600 (3)	0.0548 (14)	
C20	0.8676 (4)	0.3505 (7)	0.4120 (4)	0.060 (2)	
H20A	0.9104	0.3050	0.4336	0.072*	
C21	0.7640 (4)	0.3522 (8)	0.3232 (5)	0.075 (2)	
H21A	0.7836	0.4272	0.2951	0.112*	
H21B	0.7258	0.3843	0.3583	0.112*	
H21C	0.7384	0.2876	0.2917	0.112*	
C22	0.8521 (5)	0.1525 (7)	0.3359 (5)	0.084 (3)	
H22A	0.8067	0.0929	0.3416	0.126*	
H22B	0.8962	0.1196	0.3646	0.126*	
H22C	0.8676	0.1553	0.2857	0.126*	

### Atomic displacement parameters (Å^2^)

**Table:**

	*U*^11^	*U*^22^	*U*^33^	*U*^12^	*U*^13^	*U*^23^
Cd1	0.04830 (18)	0.02748 (15)	0.0732 (3)	0.00652 (16)	−0.0012 (3)	−0.0011 (2)
O1	0.060 (3)	0.033 (2)	0.104 (5)	0.009 (2)	0.004 (3)	−0.005 (2)
O2	0.079 (4)	0.040 (3)	0.082 (4)	0.000 (3)	0.002 (3)	−0.019 (3)
O3	0.080 (4)	0.054 (3)	0.069 (4)	0.003 (2)	−0.028 (3)	−0.015 (3)
O4	0.062 (3)	0.047 (3)	0.075 (4)	0.005 (2)	−0.024 (3)	0.003 (3)
O5	0.063 (3)	0.031 (2)	0.105 (5)	0.011 (2)	0.008 (3)	−0.005 (3)
O6	0.075 (4)	0.039 (3)	0.088 (4)	0.004 (3)	−0.011 (3)	−0.019 (3)
O7	0.070 (3)	0.048 (3)	0.067 (3)	−0.003 (2)	−0.015 (3)	−0.008 (2)
O8	0.060 (3)	0.038 (2)	0.074 (4)	0.010 (2)	−0.015 (3)	0.002 (2)
O1W	0.066 (3)	0.040 (3)	0.113 (5)	−0.001 (2)	0.022 (4)	−0.002 (3)
O2W	0.064 (3)	0.037 (2)	0.098 (4)	0.001 (2)	0.017 (3)	−0.005 (3)
N1	0.038 (3)	0.033 (2)	0.059 (4)	0.008 (2)	−0.001 (3)	0.008 (2)
N2	0.040 (2)	0.030 (2)	0.047 (3)	0.0019 (17)	−0.008 (3)	−0.007 (3)
N3	0.037 (3)	0.036 (2)	0.072 (5)	0.007 (2)	−0.002 (3)	0.012 (3)
N4	0.038 (2)	0.0281 (19)	0.059 (3)	0.0001 (17)	0.003 (3)	0.008 (3)
C1	0.037 (3)	0.031 (3)	0.052 (4)	0.002 (2)	0.004 (3)	0.000 (3)
C2	0.043 (3)	0.031 (3)	0.045 (4)	0.001 (2)	0.002 (3)	0.004 (3)
C3	0.047 (4)	0.035 (3)	0.073 (5)	−0.005 (3)	0.016 (4)	−0.007 (3)
C4	0.052 (4)	0.048 (4)	0.057 (5)	0.000 (3)	−0.010 (4)	0.008 (4)
C5	0.036 (3)	0.035 (3)	0.053 (4)	0.001 (3)	0.001 (3)	0.002 (3)
C6A	0.056 (4)	0.056 (4)	0.064 (5)	0.001 (4)	−0.011 (4)	−0.006 (4)
C7A	0.068 (5)	0.081 (5)	0.079 (5)	0.004 (4)	−0.009 (4)	−0.006 (4)
C8A	0.112 (7)	0.109 (6)	0.095 (7)	−0.021 (6)	0.006 (6)	0.017 (6)
C6B	0.061 (6)	0.064 (6)	0.069 (6)	0.002 (5)	−0.010 (5)	−0.005 (5)
C7B	0.079 (6)	0.081 (6)	0.080 (6)	−0.002 (5)	−0.007 (5)	−0.004 (5)
C8B	0.106 (9)	0.104 (9)	0.103 (9)	−0.006 (7)	−0.006 (8)	0.001 (8)
C9	0.045 (3)	0.025 (3)	0.053 (4)	−0.004 (2)	0.012 (3)	0.003 (3)
C10	0.043 (3)	0.033 (3)	0.044 (4)	0.004 (2)	0.004 (3)	0.002 (3)
C11	0.045 (4)	0.036 (3)	0.050 (4)	−0.001 (3)	0.003 (3)	−0.002 (3)
C12	0.045 (4)	0.034 (3)	0.071 (5)	−0.007 (3)	0.018 (4)	−0.007 (3)
C13	0.053 (4)	0.033 (3)	0.054 (5)	−0.002 (3)	0.005 (4)	−0.003 (3)
C14A	0.075 (5)	0.070 (5)	0.082 (5)	−0.004 (4)	−0.018 (5)	−0.009 (5)
C15A	0.108 (6)	0.119 (6)	0.097 (5)	−0.025 (5)	−0.002 (5)	−0.011 (5)
C16A	0.150 (8)	0.152 (8)	0.095 (5)	−0.016 (7)	−0.017 (6)	−0.010 (6)
C14B	0.068 (7)	0.067 (6)	0.067 (7)	−0.011 (6)	−0.012 (6)	−0.004 (6)
C15B	0.098 (6)	0.097 (6)	0.084 (6)	−0.022 (5)	−0.012 (6)	−0.008 (6)
C16B	0.122 (9)	0.114 (9)	0.102 (9)	−0.010 (8)	−0.011 (8)	−0.008 (8)
O9	0.052 (3)	0.053 (3)	0.098 (4)	−0.003 (2)	−0.003 (3)	−0.012 (3)
N5	0.045 (3)	0.050 (3)	0.059 (4)	−0.002 (3)	0.005 (3)	−0.002 (3)
C17	0.043 (4)	0.045 (4)	0.080 (6)	0.004 (3)	0.002 (4)	−0.006 (4)
C18	0.108 (6)	0.046 (4)	0.084 (6)	0.000 (4)	0.012 (5)	−0.013 (4)
C19	0.090 (6)	0.092 (6)	0.080 (7)	0.018 (5)	−0.010 (6)	−0.008 (5)
O10	0.049 (3)	0.053 (3)	0.101 (4)	−0.002 (2)	−0.013 (3)	−0.026 (3)
N6	0.056 (3)	0.051 (3)	0.058 (4)	−0.006 (3)	0.005 (3)	−0.011 (3)
C20	0.039 (3)	0.058 (4)	0.082 (6)	−0.001 (3)	−0.006 (4)	0.001 (4)
C21	0.058 (5)	0.090 (6)	0.076 (6)	0.017 (4)	−0.007 (4)	−0.019 (5)
C22	0.110 (7)	0.051 (4)	0.092 (7)	0.001 (4)	0.029 (6)	−0.011 (4)

### Geometric parameters (Å, °)

**Table:**

Cd1—N4	2.262 (4)	C7B—H7D	0.9700
Cd1—N2	2.262 (4)	C8B—H8D	0.9600
Cd1—O2W	2.325 (6)	C8B—H8E	0.9600
Cd1—O1W	2.322 (5)	C8B—H8F	0.9600
Cd1—O4	2.356 (5)	C9—C10	1.381 (7)
Cd1—O8	2.357 (5)	C9—C12	1.489 (8)
O1—C3	1.227 (7)	C10—C13	1.468 (9)
O2—C3	1.287 (8)	C11—C14B	1.490 (9)
O2—H2	0.8200	C11—C14A	1.499 (8)
O3—C4	1.269 (8)	C14A—C15A	1.555 (11)
O4—C4	1.255 (7)	C14A—H14A	0.9700
O5—C12	1.236 (7)	C14A—H14B	0.9700
O6—C12	1.275 (8)	C15A—C16A	1.467 (11)
O6—H6	0.8200	C15A—H15A	0.9700
O7—C13	1.274 (7)	C15A—H15B	0.9700
O8—C13	1.251 (6)	C16A—H16A	0.9600
O1W—H1W	0.83 (2)	C16A—H16B	0.9600
O1W—H2W	0.82 (2)	C16A—H16C	0.9600
O2W—H3W	0.80 (2)	C14B—C15B	1.537 (12)
O2W—H4W	0.80 (2)	C14B—H14C	0.9700
N1—C5	1.360 (7)	C14B—H14D	0.9700
N1—C1	1.363 (7)	C15B—C16B	1.475 (12)
N1—H1A	0.8600	C15B—H15C	0.9700
N2—C5	1.321 (7)	C15B—H15D	0.9700
N2—C2	1.376 (7)	C16B—H16D	0.9600
N3—C11	1.327 (7)	C16B—H16E	0.9600
N3—C9	1.363 (7)	C16B—H16F	0.9600
N3—H3A	0.8600	O9—C17	1.229 (6)
N4—C11	1.334 (7)	N5—C17	1.311 (7)
N4—C10	1.375 (8)	N5—C19	1.429 (9)
C1—C2	1.372 (7)	N5—C18	1.454 (6)
C1—C3	1.480 (8)	C17—H17A	0.9300
C2—C4	1.486 (9)	C18—H18A	0.9600
C5—C6B	1.490 (9)	C18—H18B	0.9600
C5—C6A	1.492 (7)	C18—H18C	0.9600
C6A—C7A	1.537 (11)	C19—H19A	0.9600
C6A—H6A	0.9700	C19—H19B	0.9600
C6A—H6B	0.9700	C19—H19C	0.9600
C7A—C8A	1.483 (10)	O10—C20	1.235 (7)
C7A—H7A	0.9700	N6—C20	1.296 (8)
C7A—H7B	0.9700	N6—C21	1.436 (9)
C8A—H8A	0.9600	N6—C22	1.455 (8)
C8A—H8B	0.9600	C20—H20A	0.9300
C8A—H8C	0.9600	C21—H21A	0.9600
C6B—C7B	1.534 (12)	C21—H21B	0.9600
C6B—H6C	0.9700	C21—H21C	0.9600
C6B—H6D	0.9700	C22—H22A	0.9600
C7B—C8B	1.476 (12)	C22—H22B	0.9600
C7B—H7C	0.9700	C22—H22C	0.9600
			
N4—Cd1—N2	178.7 (3)	N3—C9—C10	105.6 (5)
N4—Cd1—O2W	88.96 (17)	N3—C9—C12	122.3 (5)
N2—Cd1—O2W	92.14 (18)	C10—C9—C12	132.1 (6)
N4—Cd1—O1W	91.17 (18)	N4—C10—C9	108.0 (5)
N2—Cd1—O1W	87.75 (17)	N4—C10—C13	120.1 (5)
O2W—Cd1—O1W	178.6 (2)	C9—C10—C13	131.8 (6)
N4—Cd1—O4	106.78 (19)	N3—C11—N4	109.4 (5)
N2—Cd1—O4	73.93 (18)	N3—C11—C14B	123.4 (14)
O2W—Cd1—O4	92.1 (2)	N4—C11—C14B	124.6 (14)
O1W—Cd1—O4	86.57 (18)	N3—C11—C14A	125.7 (8)
N4—Cd1—O8	73.39 (19)	N4—C11—C14A	124.5 (8)
N2—Cd1—O8	105.90 (17)	O5—C12—O6	125.0 (6)
O2W—Cd1—O8	88.21 (17)	O5—C12—C9	118.2 (7)
O1W—Cd1—O8	93.1 (2)	O6—C12—C9	116.8 (6)
O4—Cd1—O8	179.7 (2)	O8—C13—O7	123.6 (6)
C3—O2—H2	109.5	O8—C13—C10	118.8 (6)
C4—O4—Cd1	114.8 (4)	O7—C13—C10	117.6 (5)
C12—O6—H6	109.5	C11—C14A—C15A	109.1 (8)
C13—O8—Cd1	114.8 (4)	C11—C14A—H14A	109.9
Cd1—O1W—H1W	117 (6)	C15A—C14A—H14A	109.9
Cd1—O1W—H2W	129 (6)	C11—C14A—H14B	109.9
H1W—O1W—H2W	104 (4)	C15A—C14A—H14B	109.9
Cd1—O2W—H3W	101 (6)	H14A—C14A—H14B	108.3
Cd1—O2W—H4W	111 (6)	C16A—C15A—C14A	110.3 (10)
H3W—O2W—H4W	109 (4)	C16A—C15A—H15A	109.6
C5—N1—C1	108.2 (4)	C14A—C15A—H15A	109.6
C5—N1—H1A	125.9	C16A—C15A—H15B	109.6
C1—N1—H1A	125.9	C14A—C15A—H15B	109.6
C5—N2—C2	107.5 (4)	H15A—C15A—H15B	108.1
C5—N2—Cd1	140.0 (4)	C11—C14B—C15B	111.2 (16)
C2—N2—Cd1	112.4 (4)	C11—C14B—H14C	109.4
C11—N3—C9	109.7 (5)	C15B—C14B—H14C	109.4
C11—N3—H3A	125.2	C11—C14B—H14D	109.4
C9—N3—H3A	125.2	C15B—C14B—H14D	109.4
C11—N4—C10	107.3 (4)	H14C—C14B—H14D	108.0
C11—N4—Cd1	139.8 (5)	C16B—C15B—C14B	109.2 (11)
C10—N4—Cd1	112.8 (4)	C16B—C15B—H15C	109.8
N1—C1—C2	106.3 (5)	C14B—C15B—H15C	109.8
N1—C1—C3	120.7 (5)	C16B—C15B—H15D	109.8
C2—C1—C3	132.9 (6)	C14B—C15B—H15D	109.8
C1—C2—N2	108.4 (5)	H15C—C15B—H15D	108.3
C1—C2—C4	131.1 (6)	C15B—C16B—H16D	109.5
N2—C2—C4	120.5 (5)	C15B—C16B—H16E	109.5
O1—C3—O2	122.6 (6)	H16D—C16B—H16E	109.5
O1—C3—C1	120.1 (7)	C15B—C16B—H16F	109.5
O2—C3—C1	117.3 (6)	H16D—C16B—H16F	109.5
O4—C4—O3	124.2 (6)	H16E—C16B—H16F	109.5
O4—C4—C2	118.2 (6)	C17—N5—C19	121.9 (6)
O3—C4—C2	117.6 (5)	C17—N5—C18	119.9 (6)
N2—C5—N1	109.5 (5)	C19—N5—C18	118.2 (6)
N2—C5—C6B	122 (2)	O9—C17—N5	125.6 (5)
N1—C5—C6B	128 (2)	O9—C17—H17A	117.2
N2—C5—C6A	127.3 (10)	N5—C17—H17A	117.2
N1—C5—C6A	123.2 (10)	N5—C18—H18A	109.5
C5—C6A—C7A	113.0 (9)	N5—C18—H18B	109.5
C5—C6A—H6A	109.0	H18A—C18—H18B	109.5
C7A—C6A—H6A	109.0	N5—C18—H18C	109.5
C5—C6A—H6B	109.0	H18A—C18—H18C	109.5
C7A—C6A—H6B	109.0	H18B—C18—H18C	109.5
H6A—C6A—H6B	107.8	N5—C19—H19A	109.5
C8A—C7A—C6A	109.6 (10)	N5—C19—H19B	109.5
C8A—C7A—H7A	109.8	H19A—C19—H19B	109.5
C6A—C7A—H7A	109.8	N5—C19—H19C	109.5
C8A—C7A—H7B	109.8	H19A—C19—H19C	109.5
C6A—C7A—H7B	109.8	H19B—C19—H19C	109.5
H7A—C7A—H7B	108.2	C20—N6—C21	120.5 (6)
C5—C6B—C7B	116 (2)	C20—N6—C22	122.7 (7)
C5—C6B—H6C	108.3	C21—N6—C22	116.8 (6)
C7B—C6B—H6C	108.3	O10—C20—N6	125.9 (6)
C5—C6B—H6D	108.3	O10—C20—H20A	117.0
C7B—C6B—H6D	108.3	N6—C20—H20A	117.0
H6C—C6B—H6D	107.4	N6—C21—H21A	109.5
C8B—C7B—C6B	110.4 (12)	N6—C21—H21B	109.5
C8B—C7B—H7C	109.6	H21A—C21—H21B	109.5
C6B—C7B—H7C	109.6	N6—C21—H21C	109.5
C8B—C7B—H7D	109.6	H21A—C21—H21C	109.5
C6B—C7B—H7D	109.6	H21B—C21—H21C	109.5
H7C—C7B—H7D	108.1	N6—C22—H22A	109.5
C7B—C8B—H8D	109.5	N6—C22—H22B	109.5
C7B—C8B—H8E	109.5	H22A—C22—H22B	109.5
H8D—C8B—H8E	109.5	N6—C22—H22C	109.5
C7B—C8B—H8F	109.5	H22A—C22—H22C	109.5
H8D—C8B—H8F	109.5	H22B—C22—H22C	109.5
H8E—C8B—H8F	109.5		

### Hydrogen-bond geometry (Å, °)

**Table:**

*D*—H···*A*	*D*—H	H···*A*	*D*···*A*	*D*—H···*A*
O2—H2···O3	0.82	1.69	2.460 (6)	155.
O6—H6···O7	0.82	1.64	2.453 (6)	174.
O1W—H1W···O10	0.83 (2)	1.94 (2)	2.763 (6)	175 (9)
O1W—H2W···O5^i^	0.82 (2)	2.00 (4)	2.771 (6)	158 (9)
O2W—H3W···O1^ii^	0.80 (2)	2.02 (3)	2.787 (6)	161 (7)
O2W—H4W···O9	0.80 (2)	2.02 (3)	2.791 (6)	162 (8)
N1—H1A···O10^iii^	0.86	1.91	2.761 (6)	170.
N3—H3A···O9^iv^	0.86	1.94	2.792 (6)	171.

Symmetry codes: (i) *x*, *y*+1, *z*; (ii) *x*, *y*−1, *z*; (iii) *x*−1/2, −*y*+3/2, *z*; (iv) *x*+1/2, −*y*+1/2, *z*.
